# Consistent individual differences in haemolymph density reflect risk propensity in a marine invertebrate

**DOI:** 10.1098/rsos.140482

**Published:** 2015-06-09

**Authors:** Ines Fürtbauer

**Affiliations:** Department of Biosciences, College of Science, Swansea University, Swansea, UK

**Keywords:** coping styles, crustaceans, haemolymph density, invertebrates, personality, physiology

## Abstract

While the literature on consistent individual differences in correlated suites of physiological and behavioural traits is steadily growing for vertebrates, invertebrates have received less attention. The few studies that do exist have measured temporary physiological states (or responses), rather than consistent individual physiological traits. Here, I explore the consistency of individual differences in physiology and behaviour of *n*=53 shore crabs (*Carcinus maenas*) by repeatedly measuring haemolymph density (HD) and the crabs' responses to a novel environment. In crustaceans, HD is directly proportional to protein concentrations, and thus indicative of physiological condition. HD was highly repeatable, and crabs showed consistent individual differences in their behavioural responses to a novel environment, thus indicating individual consistency in both physiology and behaviour. Furthermore, HD was significantly correlated with the crabs' risk propensity, i.e. individuals with higher HD spent more time near shelter. Overall, this provides the first evidence for consistency in an endogenous physiological trait in an invertebrate. The link between consistent physiology and behaviour, i.e. coping styles, analogous to those found in vertebrates, suggests metabolic and/or immunological correlates of personality which offer great potential for future studies.

## 1. Introduction

Animal personalities, i.e. consistent individual differences in behaviour, have been extensively studied in vertebrates (e.g. [1,2]) and more recently, also in invertebrates (reviewed by Mather & Logue [3] and Kralj-Fišer & Schuett [4]). In vertebrates, a large body of research has shown that consistent phenotypic differences are underpinned by consistent individual differences in physiology, e.g. physiological stress reactivity, energy metabolism, neuroendocrine characteristics and immunological reactivity (e.g. [5–7]). Such ‘coping styles’, that is consistent individual differences in correlated suites of behavioural and physiological traits [5], provide a useful concept for understanding individual adaptive capacity to deal with changes in the environment (note that, misleadingly, ‘coping style’ is sometimes used in the literature as a synonym for personality without inferring a link between physiology and behaviour).

Relationships between individual differences in behaviour and physiology have also been documented in invertebrates, and previous studies have focused on life-history variables (e.g. *Acyrthosiphon pisum* [8], *Euprymna tasmanica* [9]), parasite load (e.g. [10]), immune parameters (e.g. *Gryllus* spp. [11]), and effects of environmental conditions, e.g. food quality (e.g. *Phaedon cochleariae* [12]) or temperature (e.g. *Pagurus bernhardus* [13], *Ozius truncatus* [14]). However, in all of these examples, researchers have investigated and measured ‘temporary’ physiological states (or physiological responses), which they linked to behaviour, rather than measuring consistent individual differences in an endogenous physiological trait. This distinction between temporary states and consistent physiological traits is important because, if consistent individual differences in behaviour are promoted by a physiological trait, consistency in the physiological trait is a prerequisite. Individual consistency, or repeatability, in a trait can only be assessed by obtaining multiple measures from the same individuals (e.g. [15,16]), and to my knowledge no study has yet assessed consistent individual differences in an invertebrate endogenous physiological trait.

Here, I first investigate individual differences in the physiology of shore crabs (*Carcinus maenas*), using repeated haemolymph density (HD) measures. HD is directly proportional to protein concentrations in the blood and is commonly used as an indicator of physiological condition in crustaceans [17]. Second, I investigate individual differences in behavioural responses to a novel environment (i.e. exploration, activity and risk propensity). Finally, I test whether HD and behaviour are related.

## 2. Material and methods

### 2.1 Subjects and housing

Shore crabs (*n*=53) were collected in Swansea Bay and were sexed (*n*=33 females, *n*=20 males) based on the shape of the abdomen ([18]; electronic supplementary material, figure S1), weighed, and their carapace width (CW) measured using digital callipers (mean±s.d.: 46.2±6.7 mm). Subjects were individually marked using Tipp-Ex and were housed in a 122×61 cm aerated plastic tank, with seawater flow-through, and containing shelters. The water temperature ranged between 11.9°C and 12.4°C throughout the study. The crabs were fed mackerel or herring twice a week. The crabs were kept under standardized conditions for two months prior to any behavioural and physiological data collection to standardize recent environmental conditions.

### 2.2 Physiological and behavioural data

Once a week (*n*=6) on the same day, haemolymph was taken from the base of the fifth pereiopod (walking leg) using 21 gauge needles and 1 ml syringes. HD, i.e. the refractive index, was measured using a salinity density refractometer [17].

Behaviour in a novel environment was assessed by placing each crab in a gravel-lined test tank (W×L×H:15×54×24) containing half a flower pot as shelter (trial 1). The behaviour of the crab was recorded for 10 min using a Panasonic HDC-SD60 high-definition video camera. In order to gauge repeatability in crab behaviour, the experiment was repeated after 2 days (trial 2), with subjects being tested in a randomized order. Three behavioural measures were extracted from video: (i) *Exploration* (the percentage of test arena explored, assessed by drawing a square grid on tracking trajectories images, obtained using the EthoWatcher^®^ Tracking Module [19] and counting the number of squares (total *n*=138) visited by the crab), (ii) *Immobility* (the time spent without moving) and (iii) *Risk propensity* (the time spent near shelter, i.e. in physical contact in, under, behind, or next to it). Immobility and risk propensity were calculated using the EthoWatcher^®^ Ethography Module [19].

### 2.3 Data analysis

Parametric and non-parametric tests were conducted in SPSS Statistics 17.0. To investigate individual consistency in HD across six weeks, I calculated repeatability as an intraclass correlation coefficient (ICC) based on variance components derived from a one-way analysis of variance (ANOVA) with individual (*n*=53) as a factor [15] using the R package ICC [20]. The relationship between the two HD measures nearest to the experiments was assessed using Pearson's correlation. In order to assess sex differences in HD, a Mann–Whitney *U*-test was used. Spearman's rank correlations were used to assess (i) a potential link between body size and HD, (ii) consistency in behaviour across the two trials and (iii) relationships between the three behavioural traits. In order to test whether HD predicts behaviour, while simultaneously controlling for the effects of individual, size, sex and trial, I used linear mixed models (LMMs) using the R package *lme4* [21].

## 3. Results

### 3.1 Consistency in haemolymph density and behaviour

**Figure 1.**
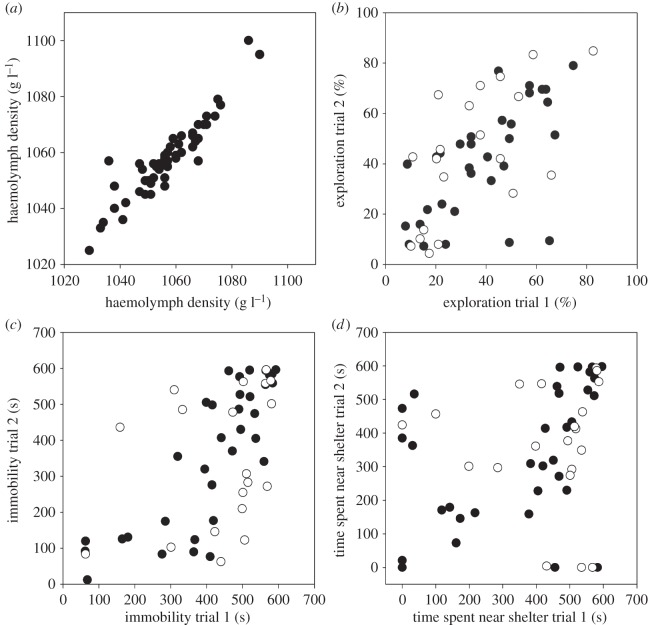
Consistency in shore crab (*a*) HD measured with a salinity density refractometer, (*b*) exploration, (*c*) immobility and (*d*) time spent near shelter (i.e. risk propensity). For (*b*,*c*,*d*), open circles represent male and filled circles female subjects (total *n*=53).

HD (average±s.d.:1058.7±12.2;range:1025−1100,*n*=53) was highly repeatable across six weeks (ICC=0.77, 95% CI=0.69, 0.84, respectively), indicating strong individual consistency. The two HD measures nearest to the behavioural trials were averaged for further analyses and were highly correlated (Pearson's *r*=0.926, *p*<0.001, *n*=53; figure 1*a*). No sex differences were found in mean HD (Mann–Whitney *U*=267, *p*=0.247). Body size and HD were unrelated (Spearman's *ρ*=−0.015, *p*=0.915, *n*=53).

Exploration, immobility and risk propensity were significantly correlated between trial 1 and trial 2 (exploration: Spearman's *ρ*=0.614, *p*<0.001, *n*=53; figure 1*b*; immobility: Spearman's *ρ*=0.663, *p*<0.001, *n*=53; figure 1*c*; risk propensity: Spearman's *ρ*=0.378, *p*=0.005, *n*=53; figure 1*d*), indicating individual consistency across trials. The three behavioural traits (averaged across trials) were also correlated (immobility and exploration: Spearman's *ρ*=−0.895, *p*<0.001; immobility and risk propensity: Spearman's *ρ*=0.552, *p*<0.001; exploration and risk propensity: Spearman's *ρ*=−0.307, *p*=0.025).

### 3.2 The link between physiology and behaviour

**Figure 2.**
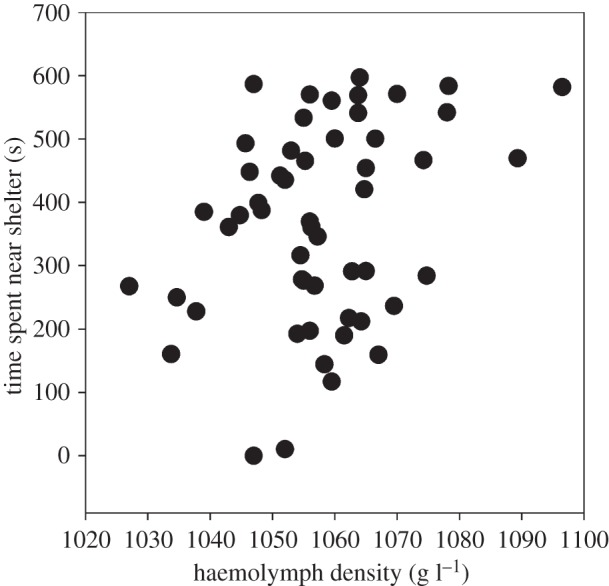
The relationship between mean HD and mean time spent near shelter in shore crabs (*n*=53). For the statistical effect of HD upon time spent near shelter (while controlling for other factors), see table 1.

HD significantly predicted risk propensity (LMM: *p*=0.009; table 1), i.e. crabs with higher HD spent more time near shelter (figure 2). Neither immobility (LMM: *p*=0.101; table 1) or exploration (LMM: *p*=0.350; table 1) were predicted by HD. No sex differences in behaviour were found (table 1). CW significantly affected exploration (LMM: *p*=0.011; table 1) and immobility (LMM: *p*=0.017; table 1) but not risk propensity (LMM: *p*=0.834; table 1). Individuals explored more (LMM: *p*=0.049) and spent less time immobile (LMM: *p*=0.002) in trial 2 than in trial 1, suggesting an effect of habituation (table 1). No difference in risk propensity was found between trials (LMM: *p*=0.277; table 1).

**Table 1.** Factors affecting exploration, immobility and risk propensity in male and female shore crabs (*n*=53). Statistically significant values are in bold.

model	predictor variable	estimate±s.e.	*p*-value
exploration	intercept	291.20±217.23	
	trial	5.24±2.59	**0.049**
	sex	−5.22±5.51	0.348
	CW	−1.04±0.39	**0.011**
	HD	−0.19±0.20	0.350
immobility	intercept	−46.48±28.46	
	trial	−1.1±0.3	**0.002**
	sex	0.87±0.72	0.234
	CW	0.13±0.10	**0.017**
	HD	0.04±0.03	0.101
risk propensity	intercept	−71.04±28.50	
	trial	−0.59±0.53	0.277
	sex	1.13±0.72	0.124
	CW	−0.01±0.05	0.834
	HD	0.07±0.03	**0.009**

## 4. Discussion

Understanding consistent individual differences in suites of correlated physiological and behavioural traits (i.e. ‘coping styles’) has been the recent focus within the animal personality framework (e.g. [5–7]). In vertebrates, individual differences in both physiology and behaviour (and links between the two) are well documented [5–7]. Conversely, in invertebrates, although consistency in behaviour, and links between physiological state and behaviour have been shown [8–14], evidence for consistent individual differences in physiology is lacking. This study demonstrates such individual consistency in physiology, i.e. HD, as well as a link between HD and risk propensity, indicative of coping styles, analogous to those observed in vertebrates (e.g. [5]).

HD in crustaceans is directly proportional to haemolymph protein levels and therefore is often used to assess physiological condition (reviewed by Lorenzon *et al*. [17]). In particular, haemolymph protein levels are used as an index of nutritional condition and are decreased in starved crustaceans (reviewed by Lorenzon *et al*. [17]). The present findings may therefore have important implications for the physiological monitoring of wild or farmed crustaceans. For instance, if individuals are not sampled repeatedly, intrinsic individual differences in haemolymph protein levels (or the HD proxy) may potentially lead to false assumptions regarding the nutritional state of the animals. More generally, the relationship between haemolymph protein and physiological state is not straightforward as total protein concentrations can vary with nutrition (see above), moulting stage, reproduction, infection, temperature, osmotic pressure, pH and salinity (reviewed by Lorenzon *et al*. [17] and Depledge & Bjerregaard [22]). In this study, housing and feeding conditions were the same for all subjects. Moulting stage, in shore crabs, is reflected in carapace coloration (green in recently moulted crabs, and red as the exoskeleton hardens [23]); however, testing green and red crabs separately revealed comparable significant results (see electronic supplementary material, table S1).

But what are the causes of individual variation in HD? In decapods, 70–95% of total haemolymph protein is haemocyanin, the oxygen transport molecule in crustaceans and many other invertebrates [22]; the remaining proteins include (among others) various antimicrobial proteins [24]. If individual differences in HD reflect individual differences in haemocyanin, this could be linked to differences in metabolic physiology. Alternatively, but not mutually exclusively, if individual differences in HD are due to differences in antimicrobial protein concentrations, this would suggest differences in immune function. These hypotheses are yet to be tested in shore crabs (or other crustaceans). Regardless, both individual differences in metabolic and immune physiology have been linked to individual differences in behaviour and, in fact, might play an important role in the link between personality and life history (e.g. [5–7,11,14]). Body-size-dependent differences in metabolism, for instance, may underlie size-dependent differences in boldness of sea anemones (*Actinia equina*) [25]. Similarly, in this study, body size affected activity, i.e. smaller crabs explored more and were less immobile, but body size and HD were unrelated. The link between body size and activity found here may have occurred owing to the ratio between crab body size and test tank size. Future studies are needed to more systematically explore how body size is linked to activity (e.g. with constant size : tank ratios, or very large test tanks).

The hypothesis that metabolism promotes phenotypic differences has received support by recent studies on ectotherms, including both vertebrates and invertebrates (e.g. [14,25,26]; see also [7]). In ectotherms, metabolism increases with temperature, and rock crabs (*Ozius truncatus*), for example, exhibit consistent individual differences in behaviour at a given temperature as well as in response to changes in temperature [14]. Biro *et al.* [14] speculate that metabolic physiology may underlie these different behavioural responses and, to further investigate this, suggest that future research should measure metabolic rates and behaviour across different temperatures. If consistent individual variation in HD is indeed linked to metabolic physiology, this study—by revealing these individual differences—provides a crucial conceptual and methodological tool for invertebrate personality research which could unravel evolutionary pathways to phenotypic differences [3,4].

## Supplementary Material

Electronic Supplementary Material S1: Risk propensity in red and green shore crabs

## Supplementary Material

Electronic Supplementary Material S2: Data

## Data accessibility

All data analysed in this study are available online in the electronic supplementary material.
