# Supplementary material for: Consistent individual differences in haemolymph density reflect risk propensity in a marine invertebrate
Source: R Soc Open Sci. 2015 Jun 9;2(6):140482. doi: 10.1098/rsos.140482 (PMC4632539; doi:10.1098/rsos.140482)
Supplement: Electronic Supplementary Material S1: Risk propensity in red and green shore crabs [file rsos140482supp1.pdf]

Electronic Supplementary Material S1: Risk propensity in red and green shore crabs

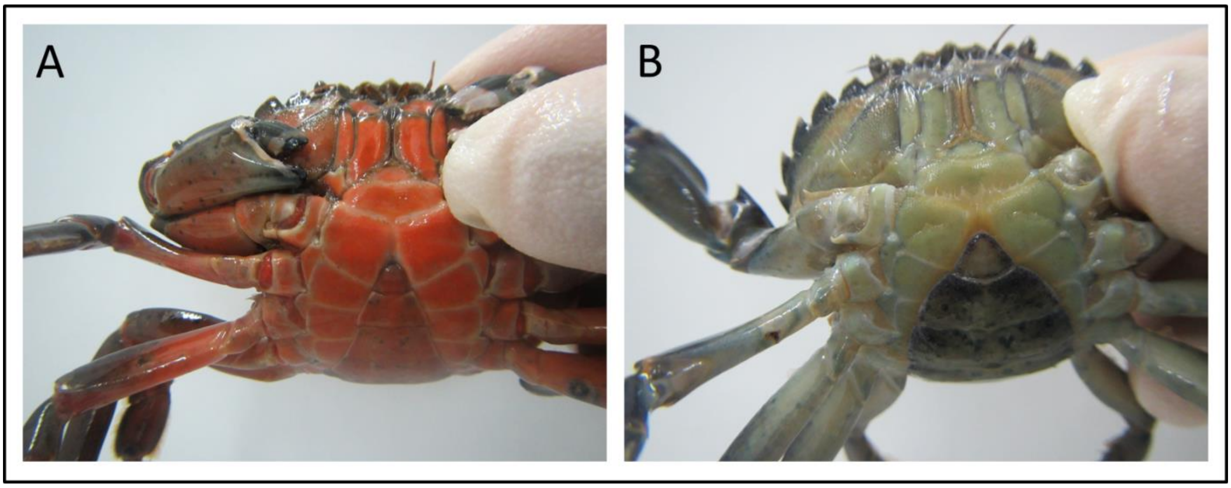

Figure S1: Ventral view of a male "red" (A) and female "green" (B) shore crab (colour reflects moulting stage and is independent of sex).

Table S1: Factors affecting risk propensity in red and green shore crabs (*Carcinus maenas*).

**Model**	**Predictor variable**	**estimate±SE**	**p-value**
red crabs	Intercept	-76.57±36.82	
n=33	Trial	-1.09±0.71	0.137
	Sex	0.50±1.05	0.638
	Carapace width	-0.03±0.08	0.652
	Haemolymph density	0.08±0.03	**0.027**
green crabs	Intercept	-125.30±42.72	
n=20	Trial	0.24±0.77	0.758
	Sex	1.08±0.82	0.205
	Carapace width	0.03±0.06	0.641
	Haemolymph density	0.12±0.04	**0.008**
